# AI Model Reporting Standards in Health Care: Systematic Review and Consolidation

**DOI:** 10.2196/92329

**Published:** 2026-10-09

**Authors:** Ekaterina Akhmad, Daniël Slob, Aiara Lobo Gomes, Simone Mingels, Andre Dekker, Johan van Soest

**Affiliations:** 1 Department of Radiation Oncology, GROW Research Institute for Oncology and Reproduction, Maastricht University Medical Centre+, Paul-Henri Spaaklaan 1, Maastricht, 6229 GS, The Netherlands, +31 (0) 43 3885457; 2 Maastro, Maastricht Radiation Oncology Institute, Maastricht, The Netherlands; 3 Brightlands Institute for Smart Society (BISS), Faculty of Science and Engineering, Maastricht University, Heerlen, The Netherlands

**Keywords:** artificial intelligence, machine learning, reporting guidelines, medicine, PRISMA, Preferred Reporting Items for Systematic Reviews and Meta-Analyses

## Abstract

**Background:**

AI technologies are currently experiencing significant growth in development and use in health care. The AI model lifecycle includes several stages, all of which should be adequately described. Reporting standards are commonly used for this purpose. However, the plain-text structure of these standards prevents transparent machine-readability of this information.

**Objective:**

This systematic review aims to investigate AI model reporting standards from clinical databases and technical documents published since 2016.

**Methods:**

The methodological systematic review was conducted according to the PRISMA (Preferred Reporting Items for Systematic Reviews and Meta-Analyses) 2020 guidelines. The inclusion criteria of this systematic review were papers published from 2016 to April 2026 in English and in a peer-reviewed journal with open access. We aimed to include standards for different data types (structured, images, and text-based). The exclusion criteria were specific medical domain, incomplete reports, and reviews. The following libraries have been consulted: Scopus and Web of Science, MEDLINE and Embase, the IEEE Xplore digital library, and the ACM Digital Library. We applied an Automated Systematic Review software tool (Utrecht University) to find relevant papers after records retrieval. Two reviewers (EA, DS) screened relevant papers independently and invited a third reviewer (JvS) to reach a consensus. All included papers were analyzed to extract relevant lifecycle stage, publication type, data type, and reporting questions. The results were presented and synthesized in a consolidated list by grouping questions and ordering them in a logical sequence according to the AI model lifecycle.

**Results:**

The total number of included studies was 25 from clinical and technical databases (22 and 3, respectively). The consolidated list includes 134 questions ordered in 7 groups: “Problem identification,” “Data collection and processing,” “Model development,” “Technical validation,” “Functional validation,” “Operation & Monitoring,” and one additional group with “Statements.” Groups according to the lifecycle stages contain all relevant information for each stage. Both clinical and technical papers reflect information for “Problem identification” (s=5), “Data collection and processing” (s=25), “Model development” (s=13), “Technical validation” (s=14). While “Functional validation” (s=30) was represented mostly in clinical papers and “Operation & Monitoring” (s=18) in technical ones. In the “Statements” (s=29), all general questions were summarized, describing reusability and reproducibility of the model, and highlighting ethical considerations, such as model fairness, explainability, interpretability, and related risk. It was shown that only 10 out of 15 FAIR (findable, accessible, interoperable, reusable) principles were addressed in the consolidated list, with focus on providing rich metadata (F2) and accurate and relevant descriptions (R1).

**Conclusions:**

We furthermore considered the mapping of the consolidated questions with FAIR principles. In future, we aim to incorporate the results of our work into a metadata schema for AI models developed according to FAIR principles.

## Introduction

### Rationale

Machine learning and AI models are currently experiencing significant growth in development and use [1], especially in health care. AI products are being increasingly deployed in hospitals worldwide [2]. The AI model lifecycle generally includes several stages, such as development, validation, and deployment [3]. All stages should be adequately described to give enough information when comparing different AI models on clinical validity, or during deployment, use, and monitoring of AI models in clinical practice. For this purpose, reporting standards are commonly used to provide reproducibility and clinical validity of findings in data science studies and specified also for AI models. Furthermore, these guidelines help authors in reporting their research, and are becoming mandatory for publishing in academic journals [4]. However, there is a rising number of standards in recent years based on the community publishing a reporting standard; from clinical to technical domains and from general to very domain specific [5].

Reporting standards mostly use approaches of storing information with plain-text and lack structure. This plain-text approach complicates comparing questions, let alone answers on the specific reporting standard questions. This reduces the comparison of AI models and methods used during the various AI model lifecycle stages. Furthermore, the paper or literature-based format of reporting prevents machine-readability. Therefore, their contents are difficult to meaningfully collect, process, and order for storing purposes in libraries, repositories, or other digital platforms. As a result, it is challenging for potential users or other stakeholders to perform systematic searches for the existence of specific AI models. The common approach to deal with these obstacles in information collection and storage is to apply the FAIR (findable, accessible, interoperable, and reusable) principles [6,7]. This framework was introduced in 2016 to describe the concept of open science and reproducible scientific practice [7]. Although originally developed for datasets, FAIR principles are also applied to AI models, since models can be considered as a number of data-containing components such as features, parameters, executable files [6,8]. In this analogy to datasets, the metadata describes the AI model, where the model itself is the data object. Several systematic reviews of reporting standards in medicine were published that aimed to provide a high-level overview for writing and reviewing research papers [9], to improve information delivery across general medical specialties [10], and to summarize the key information necessary for reporting based on the maximum agreement between standards [5]. However, we did not come across any comparison between FAIR principles and reporting standards. All stakeholders (AI developers, health care providers, clinicians, and other healthcare professionals) could benefit from using an alignment between reporting standards and FAIR principles for AI models and datasets, as provided information will be shared easily and transparently.

### Objectives

This study aimed to investigate AI model reporting standards from clinical databases and technical documents published since 2016, when the FAIR principles were introduced [7]. For that purpose, the methodological systematic review was conducted according to the PRISMA (Preferred Reporting Items for Systematic reviews and Meta-Analyses) 2020 guidelines [11]. This systematic review aimed to study the following research questions:

1. What is the complete list of themes and questions included in reporting standards?
2. How do different themes correspond with the scope of reporting standards?
3. Which of the FAIR principles are addressed in reporting standards?

## Methods

### Overview

We conducted a systematic review of published methodological journal papers and technical reports from 2016 to April 2026 to analyze the description methods of AI models in different application fields. The protocol of this review was published before at the Open Science Framework [12]. We used the PRISMA 2020 guidelines [11] to describe the results of the systematic review. The PRISMA 2020 checklist is available in the Checklist 1 .

### Eligibility Criteria

To be included in our review, a paper had to meet all the following general criteria:

- Period: from 2016 to April 2026
- Published in the English language
- Published in a peer-reviewed journal
- Open-access and/or Institutional Access

Papers that cover all data types are included, such as structured data, medical images, and text. However, we excluded papers from a specific domain (for example, medical images for surgery, biology, medical physics, nuclear medicine, and radiomics). We also excluded studies that mention only some parts of reports, for example, methods for data collection. We excluded review papers of previously published works if they did not contribute additional meaning to the reporting. For example, if the paper summarizes the previous standards without additional information. However, papers were included if it proposed a new reporting standard based on review and new information from experts.

We conducted an initial search in March 2024 to cover the period from 2016 to March 2024 and a second search in April 2026 for the period from 2024 to April 2026. Therefore, we used the same search queries for all databases and an identical analysis.

### Information Source

The general data sources included Scopus and Web of Science databases. For biomedical papers, we searched MEDLINE and Embase; for technical papers, we searched IEEE Xplore and the ACM digital libraries. For MEDLINE and Embase, we used the Ovid search interface (Ovid Technologies). The title, abstract, and keywords of papers and standard items (for example, MeSH terms), if applicable for the database, were included in search queries. The information types included articles, book chapters, books, and conference materials. In addition to the given search, we used data from systematic and narrative reviews published in peer-reviewed journals in the last 5 years [5,9,13] for validation of our search strategy and a hand search of their reference lists of included reporting papers. For the second search, we used exactly the same databases and search queries as for the first one.

### Search Strategy

The search query for each database is presented in Table S1 in Multimedia Appendix 1.

### Selection Process

We combined the identified papers in one database and then deduplicated them in EndNote (version 21.3, Clarivate) using the multifield strategy [14]. Due to the large number of records retrieved from the reference databases, we used the Automated Systematic Review software (AsReview) to find relevant papers based on their active learning algorithms [15]. The selection process was conducted for 2 searches separately.

For the first search (in March 2024), we used AsReview (version 1.6.2), then we provided prior information for the primary algorithm training, including 5 relevant records and 5 irrelevant records, and reviewed papers in 2 iterations. Firstly, a review process was performed with the default feature extraction technique, such as term frequency-inverse document frequency (TF-IDF) and a classifier (naive Bayes). One author (EA) marked the ranked articles as relevant and irrelevant, which provided continuous training for AsReview. The stopping criterion for the first version was 20 consecutive papers marked as irrelevant. After reaching the stopping criteria, the result table was extracted from AsReview and used for advanced model training on the next step. Secondly, we implemented a more advanced feature extraction technique (sentence BERT [Bidirectional Encoder Representations from Transformers]) and classifier (fully connected neural network), while using relevant and irrelevant records from the first iteration for model pretraining. As in the previous stage, the reviewer (EA) indicated papers as relevant or irrelevant, with the stopping criterion of 50 consecutive irrelevant papers. After the search update (in April 2026), we screened papers using AsReview (version 3.0.5) and a model ELAS u4, as the AsReview issued an update. We set up the stricter stopping criterion (70 consecutive papers marked as irrelevant).

After the papers’ preselection in AsReview, 2 reviewers (EA and DS) screened independently all abstracts marked as relevant to decide whether a paper met the inclusion criteria. Discussions were held to reach a consensus. Then, the same 2 reviewers screened full-text versions of papers included in the previous step and held further discussions for final consensus. A third reviewer (JvS) was invited to resolve disagreements between the 2 reviewers.

### Data Collection Process

We developed a template for data extraction in a matrix that includes rows with names of reporting questions and columns for each of the reviewing papers and the corresponding FAIR principles. All included papers were sorted randomly. The reporting topics were iteratively grouped during the review process. Questions were extracted manually from papers using the following steps:

1. Search for a table, a picture, or a structured free text that presents the proposed reporting standard.
2. Extraction of the specific reporting questions from the selected section.
3. Matching the topics with the established lifecycle stage (see below).
4. If a question matched a question already existing in the consolidated list, marked it as “Directly mentioned” (in case of complete similarity) or “Interpreting to be similar.”
5. If the question was not stated before in the consolidated list, we added it into the list and marked it as “Directly mentioned.”

We based our list of model development stages on the lifecycle published by the US Food and Drug Administration (FDA) [3], which includes the following phases:

- Planning and Design
- Data Collection and Management
- Model Building and Tuning
- Verification and Validation
- Model deployment
- Operation and Monitoring
- Real-world Performance Evaluation

To avoid misclassification of questions to lifecycle phases, the classification of reporting standard questions to a specific phase was based on the goal of the reporting standard or proposed future applications. For example, terms such as “validation” and “implementation” can have different meanings depending on context. We assumed that reporting standards mainly cover phases from 1 to 5, as most reporting standards attempt to describe the development and validation of models.

### Quality and Risk of Bias Assessment

To verify the reliability of our data extraction and analysis, 2 researchers (EA and DS) screened the papers independently. Moreover, we analyzed papers in terms of reported themes and corresponding FAIR principles. The third reviewer (JvS) contributed to reaching a consensus.

### Analysis

The reported questions from different papers were used to build a general list while matching them with corresponding stages of the AI model lifecycle. Themes were marked for the specific reviewed paper as “mentioned explicitly,” “interpreted as the same,” and none (not mentioned). We mapped extracted reporting questions with FAIR principles [16] for the AI model itself and its metadata. In our study, the AI model was assumed to be developed, so the data of the AI model will include output coefficients, weights, and other data related to the execution of the AI model. Therefore, methods regarding the training pipeline and scripts and/or decisions regarding data processing were attributed to the metadata of the AI model.

## Results

### Study Selection

A total of 25 papers [17-41] met all eligibility criteria and were included in this study. For the presentation of included papers, we used the PRISMA flow diagram [11] for systematic reviews to summarize included papers (Figure 1). We identified 19,770 papers related to reporting standards in peer-reviewed journals. After deduplicating and removing records without open access (including also institutional access), we used AsReview to screen 6166 papers at the first stage and 4027 at the second stage of review, from which we reviewed 218 full-text documents. 193 papers were excluded as they did not meet the inclusion criteria; for example, 52 papers were excluded because of the specific application domain of AI models, such as medical physics, nuclear medicine, or in-game tools.

**Figure 1.**
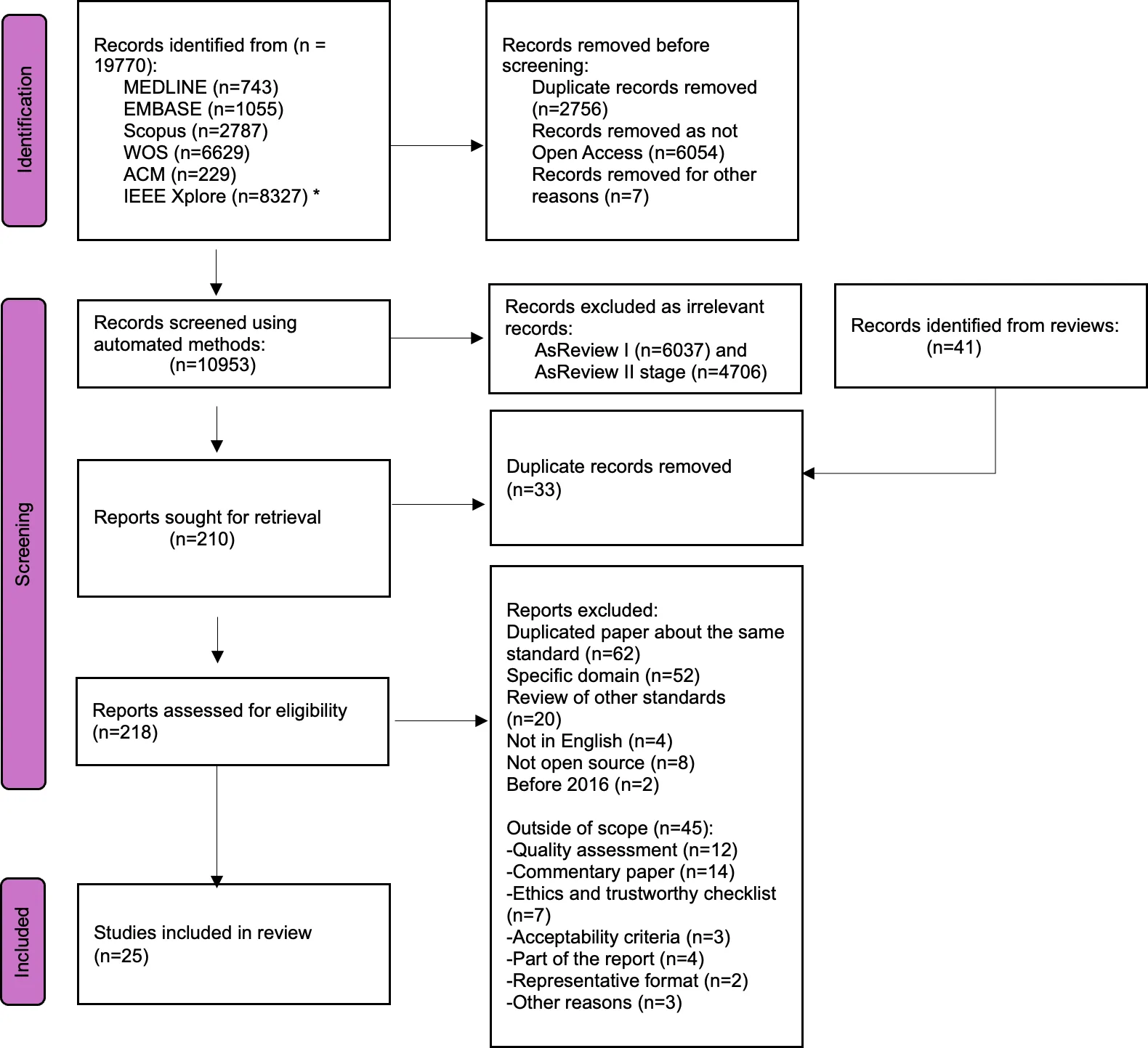
PRISMA (Preferred Reporting Items for Systematic Reviews and Meta-Analyses) Flow-diagram. AsReview: Automated Systematic Review software.

We also excluded 4 papers because they were published in other languages, 20 papers because they presented reviews of different standards without new interpretation, 8 papers had no open-access full-text, and 2 papers were published before 2016. Sixty-two records were removed due to the duplication of papers about the same reporting standard. For example, 10 papers refer to the protocol of the standard’s development. We also excluded different extensions of the same standard, such as the TRIPOD (Transparent Reporting of a Multivariable Prediction Model for Individual Prognosis or Diagnosis) guidelines, which has a few extensions or variations: TRIPOD+AI [17], TRIPOD-Cluster [42], TRIPOD for abstract [43], TRIPOD-SRMA (systematic reviews and meta-analysis) [44]. These papers would not add specifically new information to the question list (TRIPOD-Cluster aimed to describe studies based on clustered datasets, TRIPOD for abstracts focused on writing abstracts, TRIPOD-SRMA was an extension for writing systematic reviews). Only TRIPOD+AI and TRIPOD-LLM were included as the more general, which focused specifically on prediction model studies in AI or studies using large language model (LLM). We also excluded previous versions of checklists; for example, Clinical AI Modeling (CLAIM) was first published in [45] with updates in [18]. As we aim to have up-to-date questions from the working group, we included the latest version [18].

In total, 45 papers were identified as outside of scope because of different factors and were excluded from the review. These factors included, for example, whether they provide only checklists of questions for quality, ethical, and/or trustworthy assessment, not specifically for reporting. These checklists commonly include several items to assess transparency, trustworthiness, suitability, and other aspects of AI models, for example, by presenting a diagnostic quality model for integrating AI models into clinical practice [46]. As the purpose of these papers was not to report the AI studies, they were considered out of scope. Additionally, there were commentary papers and papers focusing only on the specific part of the report (eg, methods for data collection); or papers with the discussion of the representation format of the report but without describing the content itself.

### Study Characteristics

Among the included records, the number of papers from health care journals was almost 5 times more than from technical journals (22 and 3, respectively). The general characteristics of the papers included are presented in Table 1. Regarding data type, 15 papers covered reporting standards without any specific data type (general); 4 papers focused on medical images, 2 papers covered reporting of prediction models based on structured data and 4 papers were for text-based data.

**Table 1.** The characteristics of the included papers.

Group of characteristics and description	Number of records
Type of references’ source	
Clinical database	22
Technical database	3
Data type	
General	15
Medical images	4
Structured data	2
Text-based data	4
Year of publication	
2016‐2018	1
2018‐2020	2
2020‐2022	7
2022‐2024	2
2024‐2026 (April)	13

### Comparison of Reporting Standards

In total, 25 papers [17-41] were screened to fill in the template. Table 2 presents the characteristics of the included papers. From each paper, we extracted the study characteristics as the evidence that lies in the foundation of the presented question list. We could distinguish 3 main types: consensus-based research (n=14) [17-30], a scientific publication from a biomedical database (n=8) [31-38], and a scientific publication from a technical database (n=3) [39-41]. The first type was determined based on the materials and methods section in the paper, which stated that Delphi methods or other consensus-building approaches were used. The scientific publications were granted for other records published in peer-reviewed journals, but without mentioning consensus-based approaches.

**Table 2.** The characteristics of included papers.

	Citation	Data type	Study characteristics	Stage of lifecycle
1.	DECIDE-AI^a^ [19]	General	Consensus-based research	Model building and tuning, verification and validation
2.	SPIRIT-AI^b^ [20]	General	Consensus-based research	Model deployment (functional validation)
3.	CONSORT-AI^c^ [21]	General	Consensus-based research	Model deployment (functional validation)
4.	TRIPOD+AI^d^ [17]	Clinical data	Consensus-based research	Model building and tuning, verification and validation
5.	REFORMS^e^ (Kapoor et al.) [22]	General	Consensus-based research	Model building and tuning, verification and validation
6.	Luo et al [23]	Clinical data	Consensus-based research	Model building and tuning, verification and validation
7.	Tejani et al [18]	Medical images	Consensus-based research	Model building and tuning, verification and validation
8.	Moassefi et al [24]	Medical images	Consensus-based research	Model building and tuning, verification and validation
9.	CHEERS-AI^f^ (Elvidge et al) [25]	General	Consensus-based research	Model deployment (functional validation)
10.	TRIPOD-LLM^g^ (Gallifant et al) [26]	LLM^h^	Consensus-based research	Across the whole lifecycle
11.	STARD-AI^i^ (Sounderajah et al) [27]	General	Consensus-based research	Verification and validation, model deployment (functional validation)
12.	CHART^j^ [28]	LLM	Consensus-based research	Verification and validation, model deployment (functional validation)
13.	GAMER^k^ (Luo et al) [29]	LLM	Consensus-based research	Model deployment
14.	MedInAI (Bottacin et al) [30]	General	Consensus-based research	Across the whole lifecycle
15.	MI-CLAIM^l^ (Norgeot et al) [31]	Medical images	Scientific publication	Model building and tuning, verification and validation
16.	CAIR^m^ (Olczak et al) [32]	General	Scientific publication	Model building and tuning, verification and validation, model deployment
17.	Cabitza et al [33]	General	Scientific publication	Model building and tuning, verification and validation
18.	Stevens et al [34]	General	Scientific publication	Model building and tuning, verification and validation
19.	Model facts (Sendak et al) [35]	General	Scientific publication	Model deployment
20.	Kwak, Kim [36]	General	Scientific publication	Across the whole lifecycle
21.	MI-CLEAR-LLM^n^ (Park et al) [37]	LLM	Scientific publication	Model deployment
22.	RIDGE^o^ (Maleki et al) [38]	Medical images	Scientific publication	Model building and tuning, verification and validation
23.	Model card (Mitchell et al) [39]	General	Scientific publication from technical databases	Model deployment
24.	McCradden et al [40]	General	Scientific publication from technical databases	Model building and tuning, verification and validation, model deployment (functional validation)
25.	FactSheets (Arnold et al) [41]	General	Scientific publication from technical databases	Verification and validation, model deployment

a DECIDE-AI: Developmental and Exploratory Clinical Investigations of Decision Support Systems Driven by AI.

b SPIRIT-AI: Standard Protocol Items: Recommendations for Interventional Trials–AI.

c CONSORT-AI: Consolidated Standards of Reporting Trials–AI.

d TRIPOD+AI: Transparent Reporting of a Multivariable Prediction Model for Individual Prognosis or Diagnosis + AI.

e REFORMS: Consensus-based Recommendations for Machine-learning-based Science.

f CHEERS-AI: Consolidated Health Economic Evaluation Reporting Standards for Interventions that use AI.

g TRIPOD-LLM: Transparent Reporting of a Multivariable Prediction Model for Individual Prognosis or Diagnosis for studies using Large Language Models.

h LLM: large language model.

i STARD-AI: Standards for Reporting Diagnostic Accuracy–AI.

j CHART: Chatbot Assessment Reporting Tool.

k GAMER: Generative Artificial Intelligence Tools in Medical Research.

l MI-CLAIM: Minimum Information about Clinical AI Modeling.

m CAIR: Clinical AI Research.

n MI-CLEAR-LLM: Minimum Reporting Items for Clear Evaluation of Accuracy Reports of Large Language Models in Health care.

o RIDGE: Reproducibility, Integrity, Dependability, Generalizability, and Efficiency.

Papers that were intended to guide the reporting aspects for clinical trials of AI models ([Standard Protocol Items: Recommendations for Interventional Trials–AI] SPIRIT-AI [20] and (Consolidated Standards of Reporting Trials–AI) CONSORT-AI [21]) or health economic evaluations ([Consolidated Health Economic Evaluation Reporting Standards for Interventions that use AI] CHEERS-AI [25]) were related more to the functional validation that occurs before market certification. This process could be connected with the phase of “Model deployment,” as the AI model at this stage has a scalable and capable format of integration into a system and could be validated in the real clinical practice workflow. Some papers provide only a brief explanation, for example, Model facts [35] and Model cards [39]; in these cases, the stage was defined according to the questions.

The consolidated list was formed by grouping 134 questions and ordering them in a logical sequence according to the AI model lifecycle. We extracted 7 groups of questions “Problem identification,” “Data collection and processing,” “Model development,” “Technical validation,” “Functional validation,”, “Operation & Monitoring,” and one additional group with “Statements.” For cases when one question relates to several questions from the consolidated list, we matched them correspondingly. The complete consolidated list of questions can be found in Multimedia Appendix 2. We will discuss in detail each of the formed groups in the Problem Identification section.

### Problem Identification

Figure 2 shows questions that were extracted for the problem identification stage (s=5) that could be reflected in the introduction section of the article, containing also information about the title and objectives of the study. Since problem identification is seen as the most important stage in the (machine learning) ML lifecycle, reporters should address extracted questions about the clinical problem, intended use, and the rationale for the ML model development. This diagram shows that almost all papers cover these questions to some extent, except the papers by Moassefi et al [24] and Generative Artificial Intelligence Tools in Medical Research (GAMER) [29]. The maximum number of questions for problem identification (14 items) was found in the paper by Luo et al [23], which covered detailed questions about—for example—the prediction goal and the prediction model type (such as classification, regression, and survival prediction).

**Figure 2.**
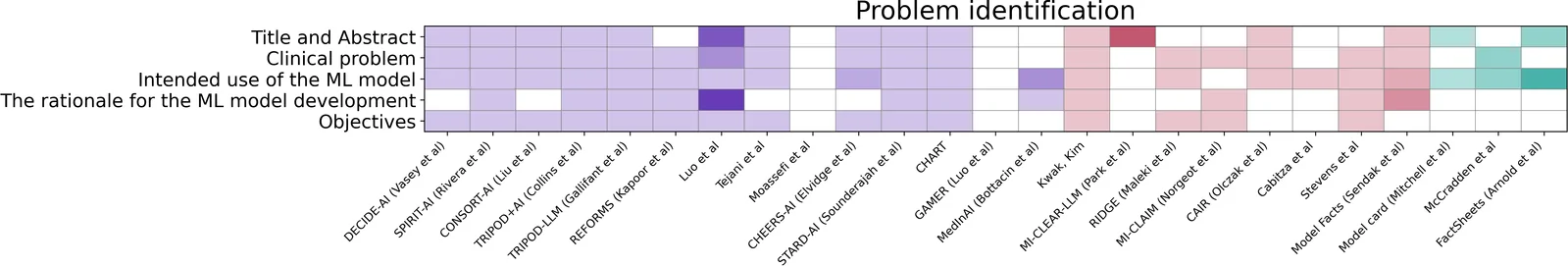
Questions from the consolidated list that relate to the problem identification. The color code provides the information about evidence characteristics: purple—consensus-based research, red—scientific publications, green—scientific publication from technical databases. The color intensity corresponds to the number of questions addressed in each paper, with the highest intensity for the maximum number [17-27,29-41]. CAIR: Clinical AI Research; CHART: Chatbot Assessment Reporting Tool; CHEERS-AI: Consolidated Health Economic Evaluation Reporting Standards for Interventions that use AI; CONSORT-AI: Consolidated Standards of Reporting Trials–AI; DECIDE-AI: Developmental and Exploratory Clinical Investigations of Decision Support Systems Driven by Artificial Intelligence; GAMER: Generative Artificial Intelligence Tools in Medical Research; MI-CLAIM: Minimum Information About Clinical AI Modeling; MI-CLEAR-LLM: Minimum Reporting Items for Clear Evaluation of Accuracy Reports of Large Language Models in Health care; ML: machine learning; REFORMS: Consensus-based Recommendations for Machine-learning-based Science; RIDGE: Reproducibility, Integrity, Dependability, Generalizability, and Efficiency; SPIRIT-AI: Standard Protocol Items: Recommendations for Interventional Trials–AI; STARD-AI: Standards for Reporting Diagnostic Accuracy–AI; TRIPOD+AI: Transparent Reporting of a Multivariable Prediction Model for Individual Prognosis or Diagnosis + AI; TRIPOD-LLM: Transparent Reporting of a Multivariable Prediction Model for Individual Prognosis or Diagnosis for studies using Large Language Models.

### Model Development

Figure 3 represents questions for the model development phase (s=13), ranging from the list of all training datasets to the hardware and packages used. Only SPIRIT-AI and CONSORT-AI do not require this information, as they were aimed at clinical evaluation of the AI model as a final product. The most frequent questions relate to the model type and training and tuning process with different levels of detail. For example, some papers used the general expressions like “How were the models trained” and “Information about training algorithms,” while Moassefi et al [24] and Olczak et al [32] ask for specific details. We extracted the question “Tools to verify the performance metrics” mentioned only in 1 paper by Mitchel et al [39] and “Decision thresholds” in 2 papers by Arnold et al [41] and STARD-AI [27]. We included the specific description for all questions in the Multimedia Appendix 2.

**Figure 3.**
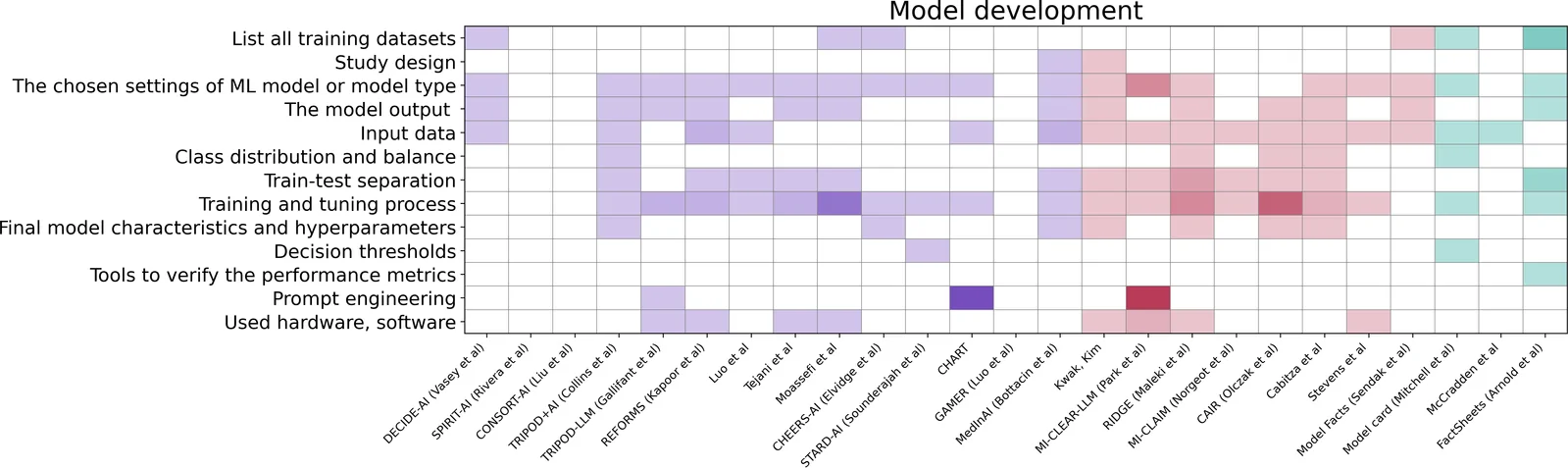
Questions from the consolidated list that relate to the model development. The color code provides the information about evidence characteristics: purple—consensus-based research, red—scientific publications, green—scientific publication from technical databases. The color intensity corresponds to the number of questions addressed in each paper, with the highest intensity for the maximum number [17-27,29-41].CAIR: Clinical AI Research; CHART: Chatbot Assessment Reporting Tool; CHEERS-AI: Consolidated Health Economic Evaluation Reporting Standards for Interventions that use AI; CONSORT-AI: Consolidated Standards of Reporting Trials–AI; DECIDE-AI: Developmental and Exploratory Clinical Investigations of Decision Support Systems Driven by Artificial Intelligence; GAMER: Generative Artificial Intelligence Tools in Medical Research; MI-CLAIM: Minimum Information About Clinical AI Modeling; MI-CLEAR-LLM: Minimum Reporting Items for Clear Evaluation of Accuracy Reports of Large Language Models in Health care; ML: machine learning; REFORMS: Consensus-based Recommendations for Machine-learning-based Science; RIDGE: Reproducibility, Integrity, Dependability, Generalizability, and Efficiency; SPIRIT-AI: Standard Protocol Items: Recommendations for Interventional Trials–AI; STARD-AI: Standards for Reporting Diagnostic Accuracy–AI; TRIPOD+AI: Transparent Reporting of a Multivariable Prediction Model for Individual Prognosis or Diagnosis + AI; TRIPOD-LLM: Transparent Reporting of a Multivariable Prediction Model for Individual Prognosis or Diagnosis for studies using Large Language Models.

### Technical Validation

Figure 4 demonstrates the reporting questions (s=14) for the technical validation of AI models. This testing can be performed on internal or external datasets. The goal of this lifecycle stage is to assess the technical performance of the AI model. The general topics, such as methods (n=17 ) [17,18,22-27,31-34,36,37,39-41], general results (n=19) [17-19,22-27,30-41], and results across clusters and subgroups (n=10) [17,18,23,24,33,35,36,38-40], were addressed in most papers. Fewer papers (n=6 [30,31,33,36,40,41] and n=5 [30,31,36,38,41]) cover the remaining questions, such as model bias assessment and testing for data shift and drift detection. Several standards mentioned the question “Comparison against appropriate baselines” aimed to show an analysis of received validation results against appropriate baselines. The baseline values are usually stated from the literature, for example, from validation studies of similar AI models.

**Figure 4.**
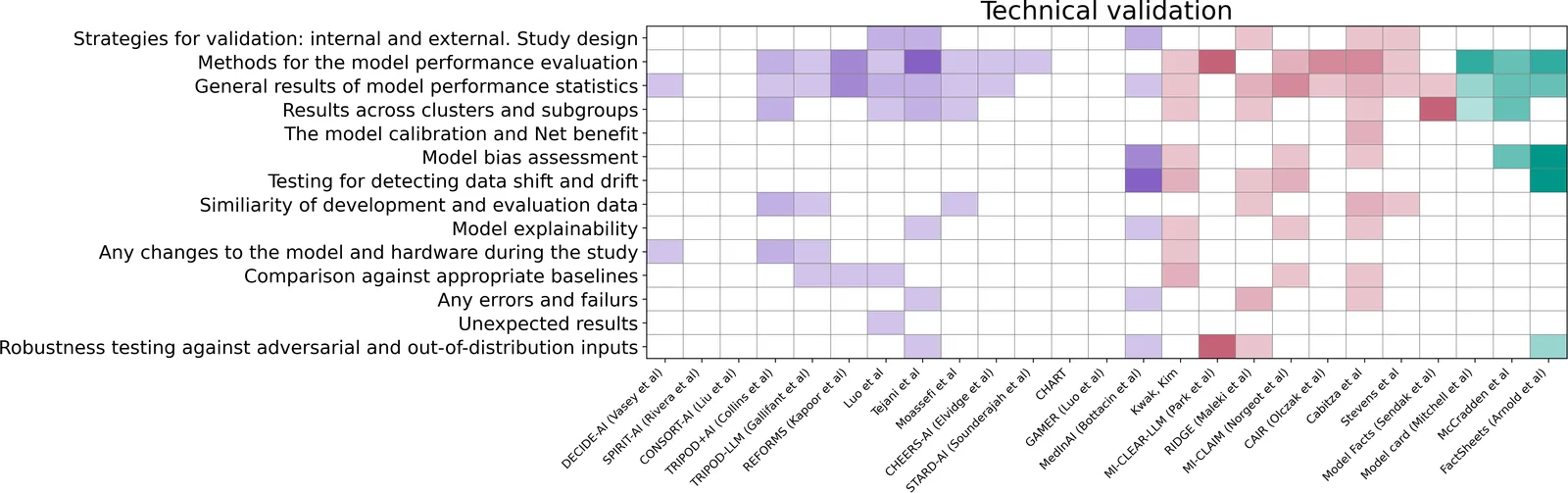
Questions from the consolidated list that relate to the technical validation. The color code provides the information about evidence characteristics: purple—consensus-based research, red—scientific publications, green—scientific publication from technical databases. The color intensity corresponds to the number of questions addressed in each paper, with the highest intensity for the maximum number [17-27,29-41]. CAIR: Clinical AI Research; CHART: Chatbot Assessment Reporting Tool; CHEERS-AI: Consolidated Health Economic Evaluation Reporting Standards for Interventions that use AI; CONSORT-AI: Consolidated Standards of Reporting Trials–AI; DECIDE-AI: Developmental and Exploratory Clinical Investigations of Decision Support Systems Driven by Artificial Intelligence; GAMER: Generative Artificial Intelligence Tools in Medical Research; MI-CLAIM: Minimum Information About Clinical AI Modeling; MI-CLEAR-LLM: Minimum Reporting Items for Clear Evaluation of Accuracy Reports of Large Language Models in Health care; ML: machine learning; REFORMS: Consensus-based Recommendations for Machine-learning-based Science; RIDGE: Reproducibility, Integrity, Dependability, Generalizability, and Efficiency; SPIRIT-AI: Standard Protocol Items: Recommendations for Interventional Trials–AI; STARD-AI: Standards for Reporting Diagnostic Accuracy–AI; TRIPOD+AI: Transparent Reporting of a Multivariable Prediction Model for Individual Prognosis or Diagnosis + AI; TRIPOD-LLM: Transparent Reporting of a Multivariable Prediction Model for Individual Prognosis or Diagnosis for studies using Large Language Models.

### Functional Validation

As shown in Figure 5, questions related to the functional validation (s=30) were primarily mentioned in 4 papers: (Developmental and Exploratory Clinical Investigations of Decision Support Systems Driven by Artificial Intelligence) DECIDE-AI, SPIRIT-AI, CONSORT-AI, and CHEERS-AI. DECIDE-AI describes the early-stage clinical evaluation of an AI model. Evaluation of AI model real-world effectiveness and safety during clinical trials is reported by standards for trial protocols (SPIRIT-AI) and trial reports (CONSORT-AI). These standards include a broad spectrum of questions about studying primary outcomes of AI intervention and any observed harm or risks to patient safety. CHEERS-AI is used for reporting economic evaluations of AI-based health interventions and measuring cost-effectiveness. In addition, the work of Kwak and Kim [36] presents a reporting guideline and a checklist across diverse study designs.

**Figure 5.**
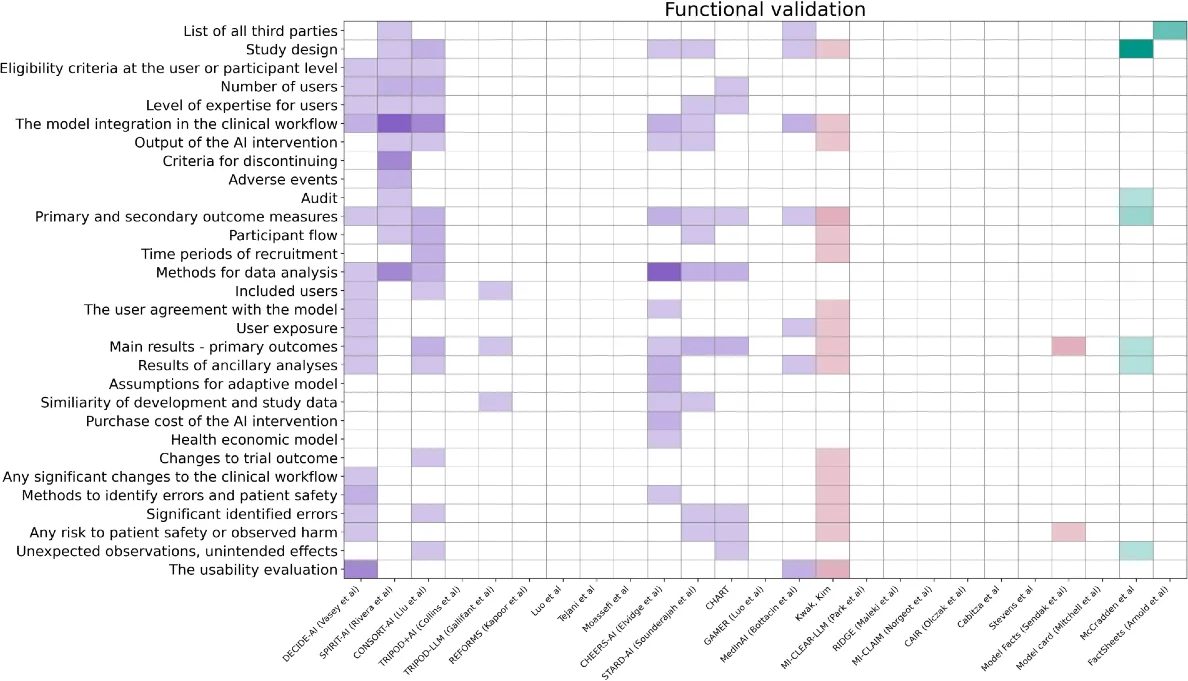
Questions from the consolidated list that relate to the functional validation. The color code provides the information about evidence characteristics: purple—consensus-based research, red—scientific publications, green—scientific publication from technical databases. The color intensity corresponds to the number of questions addressed in each paper, with the highest intensity for the maximum number [17-27,29-41]. CAIR: Clinical AI Research; CHART: Chatbot Assessment Reporting Tool; CHEERS-AI: Consolidated Health Economic Evaluation Reporting Standards for Interventions that use AI; CONSORT-AI: Consolidated Standards of Reporting Trials–AI; DECIDE-AI: Developmental and Exploratory Clinical Investigations of Decision Support Systems Driven by Artificial Intelligence; GAMER: Generative Artificial Intelligence Tools in Medical Research; MI-CLAIM: Minimum Information About Clinical AI Modeling; MI-CLEAR-LLM: Minimum Reporting Items for Clear Evaluation of Accuracy Reports of Large Language Models in Health care; ML: machine learning; REFORMS: Consensus-based Recommendations for Machine-learning-based Science; RIDGE: Reproducibility, Integrity, Dependability, Generalizability, and Efficiency; SPIRIT-AI: Standard Protocol Items: Recommendations for Interventional Trials–AI; STARD-AI: Standards for Reporting Diagnostic Accuracy–AI; TRIPOD+AI: Transparent Reporting of a Multivariable Prediction Model for Individual Prognosis or Diagnosis + AI; TRIPOD-LLM: Transparent Reporting of a Multivariable Prediction Model for Individual Prognosis or Diagnosis for studies using Large Language Models.

Model Facts consolidate general information about patient safety and efficacy evaluation, which is why we also present it in this section. McCradden et al [40] explores ethical and social justice principles not only for model development, but also for model evaluation, including silent trial evaluation (prospective non-interventional evaluation) and prospective clinical evaluation to study the influence of the AI intervention on clinical decision-making. However, it focuses more on ethical considerations and the importance of diversity in AI clinical trials rather than other aspects of reports.

Figure 6 represents questions for the Operation and Monitoring stage of the AI system (s=18). Arnold et al [41] (FactSheets) is the most complete reporting standard in this domain, listing generic technical descriptions that are needed for the ML model operation stage. Some questions are addressed by commonly used Model card [39] and Model Facts [35]. MedinAI includes several sections to describe an AI model as service that should be technically approved and effective in real-world health care. In addition, it also provides a separate group of model updating items [30]. CHART [28] and GAMER [29] relate to the use of LLM models.

**Figure 6.**
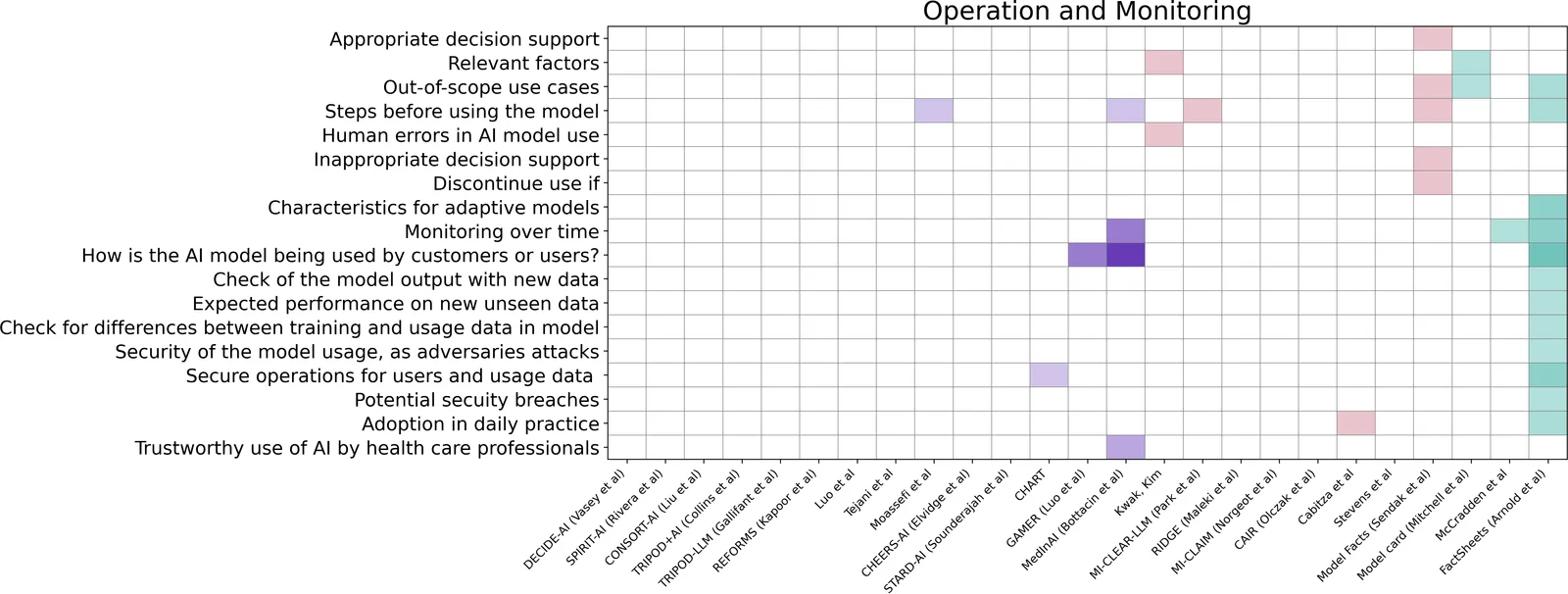
Questions from the consolidated list that relate to the operation and monitoring. The color code provides the information about evidence characteristics: purple—consensus-based research, red—scientific publications, green—scientific publication from technical databases. The color intensity corresponds to the number of questions addressed in each paper, with the highest intensity for the maximum number [17-27,29-41]. CAIR: Clinical AI Research; CHART: Chatbot Assessment Reporting Tool; CHEERS-AI: Consolidated Health Economic Evaluation Reporting Standards for Interventions that use AI; CONSORT-AI: Consolidated Standards of Reporting Trials–AI; DECIDE-AI: Developmental and Exploratory Clinical Investigations of Decision Support Systems Driven by Artificial Intelligence; GAMER: Generative Artificial Intelligence Tools in Medical Research; MI-CLAIM: Minimum Information About Clinical AI Modeling; MI-CLEAR-LLM: Minimum Reporting Items for Clear Evaluation of Accuracy Reports of Large Language Models in Health care; ML: machine learning; REFORMS: Consensus-based Recommendations for Machine-learning-based Science; RIDGE: Reproducibility, Integrity, Dependability, Generalizability, and Efficiency; SPIRIT-AI: Standard Protocol Items: Recommendations for Interventional Trials–AI; STARD-AI: Standards for Reporting Diagnostic Accuracy–AI; TRIPOD+AI: Transparent Reporting of a Multivariable Prediction Model for Individual Prognosis or Diagnosis + AI; TRIPOD-LLM: Transparent Reporting of a Multivariable Prediction Model for Individual Prognosis or Diagnosis for studies using Large Language Models.

### Statements

In the section “Statements” (Figure 7), we summarize all questions (s=29) with general model information, supporting its use in clinical practice. In addition, this section includes topics regarding the reusability and reproducibility of the model, such as data availability and code sharing, license, and information about protocol registration and ethics approval. Other questions reflect model fairness, explainability and interpretability, related risk and other ethical considerations, that are mentioned at various stages of AI lifecycle.

**Figure 7.**
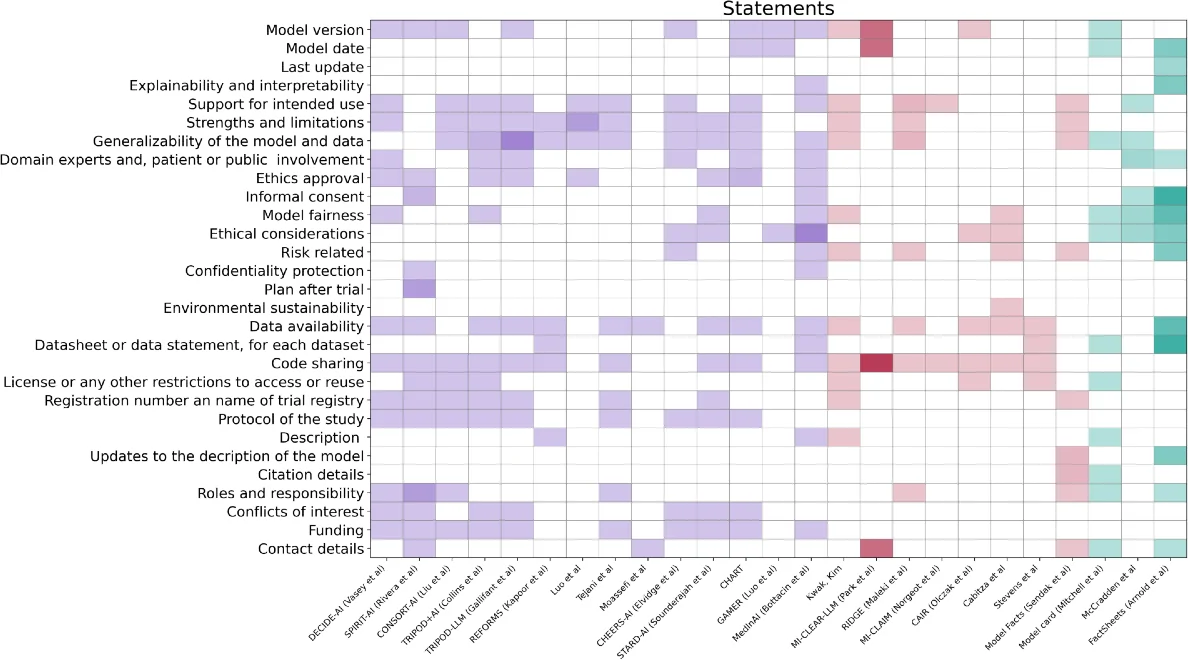
Questions from the consolidated list that relate to the statements about the model. The color code provides the information about evidence characteristics: purple—consensus-based research, red—scientific publications, green—scientific publication from technical databases. The color intensity corresponds to the number of questions addressed in each paper, with the highest intensity for the maximum number [17-27,29-41]. CAIR: Clinical AI Research; CHART: Chatbot Assessment Reporting Tool; CHEERS-AI: Consolidated Health Economic Evaluation Reporting Standards for Interventions that use AI; CONSORT-AI: Consolidated Standards of Reporting Trials–AI; DECIDE-AI: Developmental and Exploratory Clinical Investigations of Decision Support Systems Driven by Artificial Intelligence; GAMER: Generative Artificial Intelligence Tools in Medical Research; MI-CLAIM: Minimum Information About Clinical AI Modeling; MI-CLEAR-LLM: Minimum Reporting Items for Clear Evaluation of Accuracy Reports of Large Language Models in Health care; REFORMS: Consensus-based Recommendations for Machine-learning-based Science; RIDGE: Reproducibility, Integrity, Dependability, Generalizability, and Efficiency; SPIRIT-AI: Standard Protocol Items: Recommendations for Interventional Trials–AI; STARD-AI: Standards for Reporting Diagnostic Accuracy–AI; TRIPOD+AI: Transparent Reporting of a Multivariable Prediction Model for Individual Prognosis or Diagnosis + AI; TRIPOD-LLM: Transparent Reporting of a Multivariable Prediction Model for Individual Prognosis or Diagnosis for studies using Large Language Models.

### Data Collection and Processing

Finally, steps for data collection and processing were included in the last section of questions (Figure 8). This section contains patient and data description questions (s=25), including data collection and measurement techniques, as well as questions about data preprocessing and analysis, and handling of poor-quality and missing data. Only 3 papers [30, 36, 18] included requirements for deidentification methods, as it was related to medical images which require more proficient methods to deidentify personal information from DICOM (Digital Imaging and Communications in Medicine) tags. We could not distinguish between questions for clinical and technical papers.

**Figure 8.**
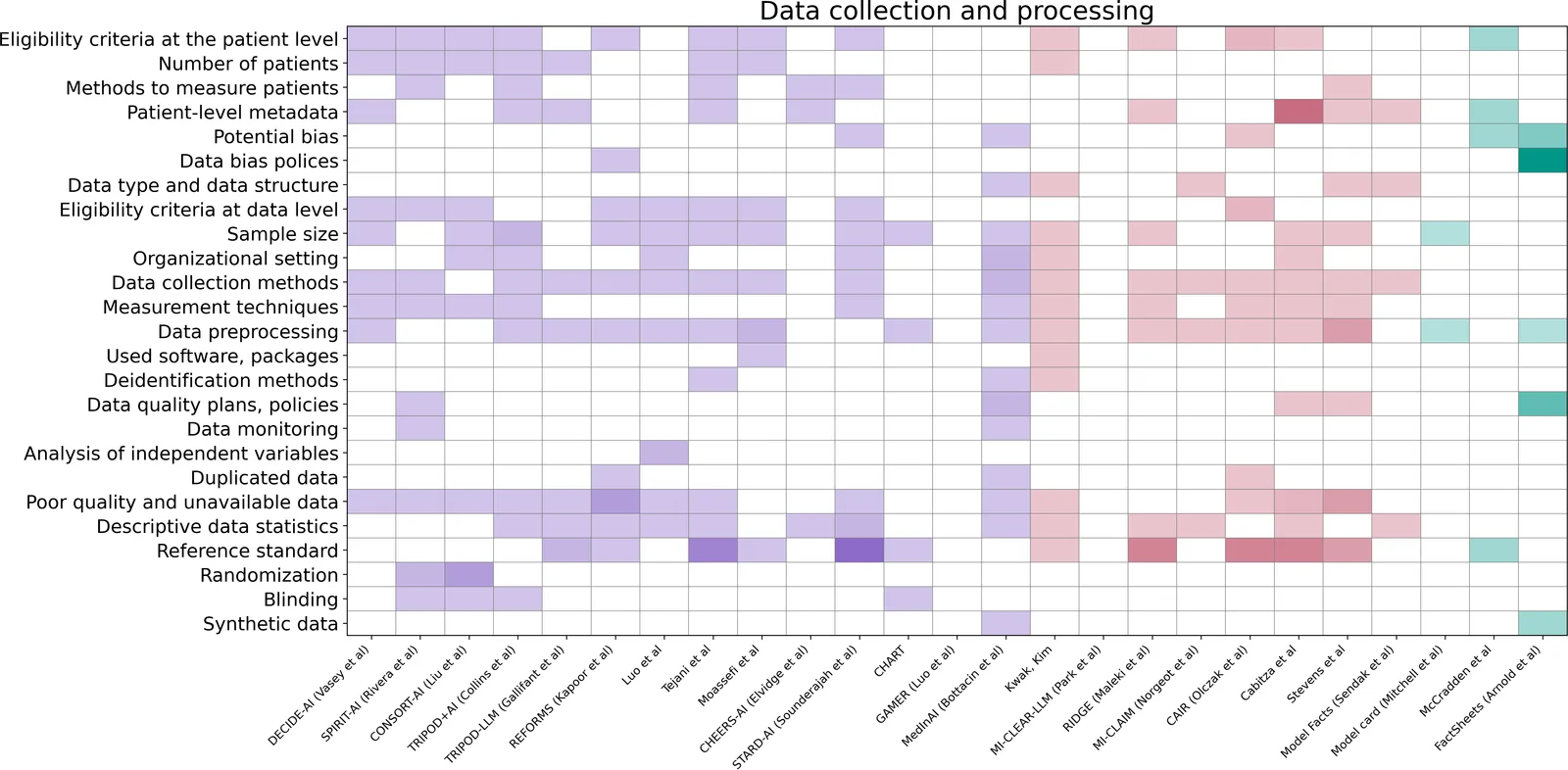
Questions from the consolidated list that relate to the data collection and processing. The color code provides the information about evidence characteristics: purple – consensus-based research, red – scientific publications, green – scientific publications from technical databases. The color intensity corresponds to the number of questions addressed in each paper, with the highest intensity for the maximum number [17-27,29-41]. CAIR: Clinical AI Research; CHART: Chatbot Assessment Reporting Tool; CHEERS-AI: Consolidated Health Economic Evaluation Reporting Standards for Interventions that use AI; CONSORT-AI: Consolidated Standards of Reporting Trials–AI; DECIDE-AI: Developmental and Exploratory Clinical Investigations of Decision Support Systems Driven by Artificial Intelligence; GAMER: ; MI-CLAIM: Minimum Information About Clinical AI Modeling; MI-CLEAR-LLM: Minimum Reporting Items for Clear Evaluation of Accuracy Reports of Large Language Models in Health care; REFORMS: ; RIDGE: Reproducibility, Integrity, Dependability, Generalizability, and Efficiency; SPIRIT-AI: Standard Protocol Items: Recommendations for Interventional Trials–AI; STARD-AI: Standards for Reporting Diagnostic Accuracy–AI; TRIPOD+AI: Transparent Reporting of a Multivariable Prediction Model for Individual Prognosis or Diagnosis + AI; TRIPOD-LLM: Transparent Reporting of a Multivariable Prediction Model for Individual Prognosis or Diagnosis for studies using Large Language Models.

### Reporting Standards and FAIR Principles

We matched the FAIR principles with the sections or phases as explained above by using the general FAIR principles published by Wilkinson et al in 2016 [7]. These recommendations cover both metadata and data (in our case, the AI model).

Figure 9 shows the connection between question groups – as discussed in the previous sections – and FAIR principles. It was shown that only 10 out of 15 FAIR principles were addressed in the consolidated list. Multimedia Appendix 2 includes the whole list of reporting questions together with corresponding FAIR principles. In this section, we are discussing only general findings related to extracted groups of questions.

**Figure 9.**
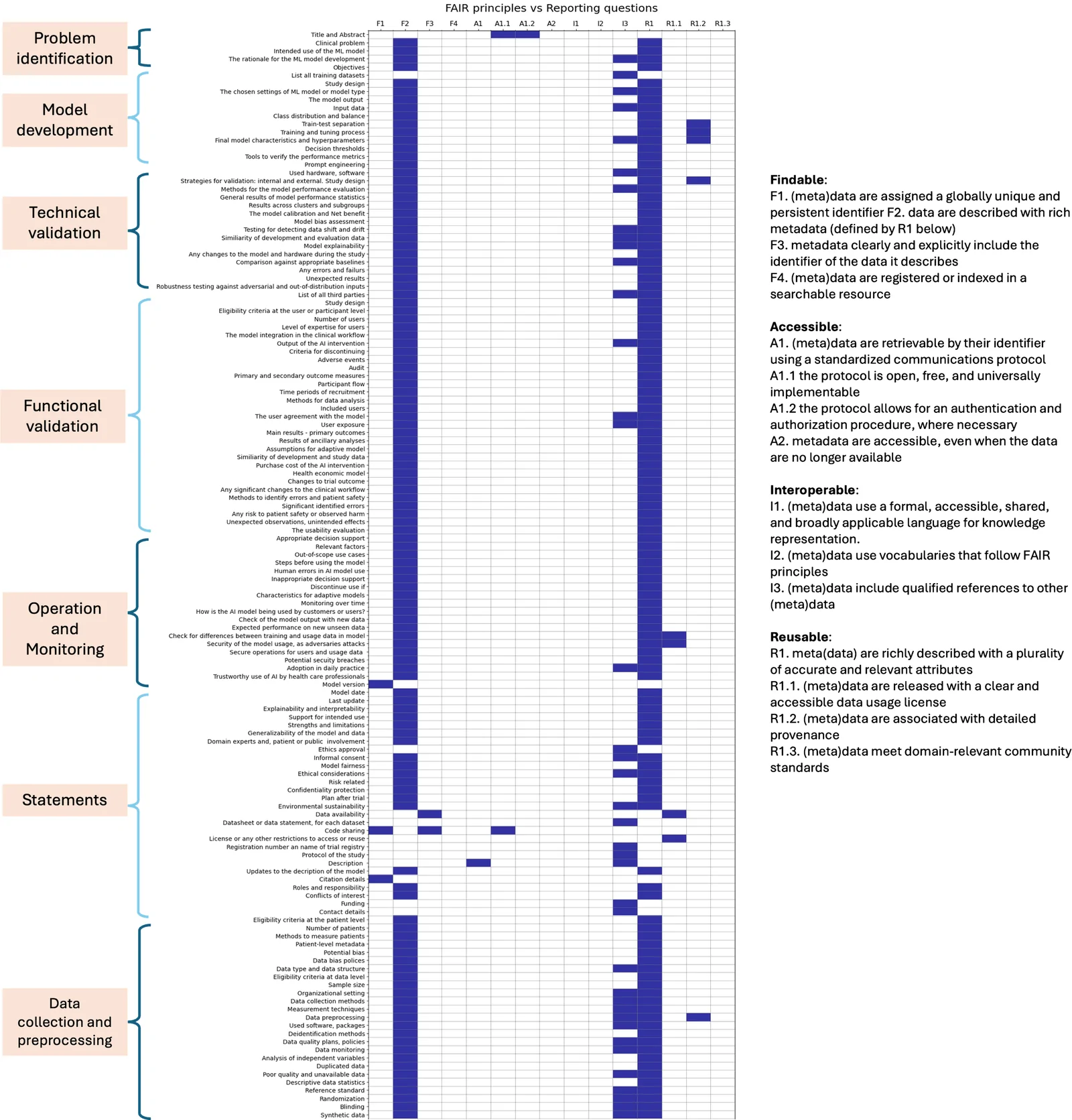
The mapping of reporting questions to FAIR principles. FAIR: findable, accessible, interoperable, reusable; ML: machine learning; ML: machine learning.

We assumed that by answering reporting questions for all question groups, an AI model should be described with rich metadata (FAIR principle: F2) that includes references to other metadata (I3) and contains accurate and relevant descriptions of all required processes (R1). As some of the reporting standards were related to paper submissions, we assume that these papers would have a DOI or another identifier that will be accessible even if the model is no longer available (A2). Although we must acknowledge that scientific papers are not in a machine-readable format.

Two question groups, “Data collection & processing” and “Model building & tuning,” contain information about specific processes such as the formation of datasets for training or validations and training itself. These descriptions of all manipulations might be relevant as provenance information (R1.2). Only 1 paper, Cabitza et al [33], contains information about the terminology standards (LOINC (Logical Observation Identifiers Names and Codes), ICD-11 (International Classification of Diseases, eleventh revision), SNOMED CT (Systematized Nomenclature of Medicine – Clinical Terms)) as a part of input data description. As it was not common for all standards, we could not mention it in the list of applied FAIR principles; however, it might potentially relate to vocabularies that follow FAIR principles (I2) and meeting domain-relevant community standards (R1.3).

The group of questions named “General model statements” relates to the principles F2, I3, A2, and R1. With questions about code and dataset sharing, we assume providing the links using secure transfer protocols (A1) that are open, free, and universally implementable (A1.1). Security operations and contact details provide information about access to the model and datasets that could be aligned with the authentication (A1.2). The question about license and restriction on how to access or reuse the model relates to the R1.1 principle. Finally, items about funding, ownership, and contact details could also be associated with R1.2 as additional provenance information.

## Discussion

### General interpretation

The paper studied published reporting standards for AI models from both clinical and technical perspectives to extract and consolidate the complete list of reporting questions, and assess whether they aligned with FAIR principles. This systematic review analyzed 25 reporting standards for AI models. The results of our review present the complete list of reporting questions related to different stages of the lifecycle, mapped to FAIR principles. Readers could report AI studies at different lifecycle stages using a more comprehensive consolidated reporting guideline collected in this systematic review.

The previous study by Klement et al [47] showed the 37 extracted reporting items grouped into the 5 categories: study details, data description, methodology for model development, model evaluation, and explainability. The authors consolidated items from specific domains (like biology, nuclear medicine, and cardiovascular diseases) and also some not finalized checklists (such as MINIMAR [Minimum Information for Medical AI Reporting], as only a development protocol was published [48]). Since the authors included standards only from clinical sources, the final list does not incorporate details about technical validation, deployment, operation, and monitoring stages, as presented in our review. Another paper was published by Kolbinger et al [5] where the list of reporting topics was combined in 1 set and grouped into 5 categories: clinical rationale, data, model training and validation, critical appraisal, ethics, and reproducibility. We observed the overlap with our extracted list, with more detailed questions included in the model training, and a separate section for 2 types of validation: technical and functional. We also extracted a more extended list of statements that relate to critical appraisal, ethics, and reproducibility groups. The final list was also formed considering that at least 50% of all standards reported this question. We did not use any thresholds for consolidation as we wanted to see how different questions aligned with lifecycle stages, and all reporting standards served different purposes.

Another approach was used in the paper of Shiferaw et al [49], in which the authors discussed the quality of published reporting standards and incorporated the necessary topics related to transparency, reproducibility, and ethics in medical AI research. The research identified that TRIPOD+AI, DECIDE-AI, APPRAISE-AI (Assessment of Predictive Performance and Bias in Artificial Intelligence Studies), SPIRIT-AI, and CONSORT-AI have the highest score in quality evaluation. We included these papers except for APPRAISE-AI, which matched the exclusion criteria as a paper from a specific domain. In the other review papers, Klontzas et al [50] proposed a consolidated list for medical radiological images that could be used during the review process of studies, while Garbin and Marques [51] highlighted the exact purpose by aligning the reporting standards with the AI model lifecycle stage while not proposing a consolidated list of standards. The stages used are overlapping with ours, except that it has one stage called “Product test” without distinguishing between technical and functional validations. In our opinion, this distinction is important since these validations are performed by different specialists and will produce different validation properties and results. Finally, Ibrahim et al [13] reviewed reporting standards for different purposes, such as clinical trials and diagnostic accuracy studies. This paper does not provide a consolidated list and focuses on clinical implications.

### Findings According to the AI Model Lifecycle

As we consolidated the list of questions on what to report on each stage of the AI model lifecycle, we need to discuss its interpretation. The lifecycle typically initiates by defining the problem. Most of the papers ask it in a general way, for example, to describe the clinical background, the intended use of the ML model, and the rationale for the ML model development. Luo et al [23] included a separate section describing the setting of the study and the prediction problem. These questions could provide comprehensive information about the intentions behind the proposed ML model development and propagate into specific inclusion and exclusion criteria for model use cases. Most of the included standards stated the necessity to report this information.

The group “Data collection and management” includes a broad spectrum of questions related to patient and data eligibility criteria, as well as methods for collection and measurement. These questions can be repeated for different stages of the AI lifecycle – for training, technical, and functional validation. Data collection was covered by most included standards and highlighted the importance of these questions for all stages of the lifecycle, which is characterized using datasets. Previously, a datasheet was developed to elicit the information that a dataset might contain [52]. This proposal also matched the key stages of the dataset lifecycle. In our work, we did not compare questions from the consolidated list with the datasheet, as it was not included by our eligibility criteria. Although we can expect a considerable overlap between items. Arnold et al mentioned that the datasheet found a significant reflection in the Factsheet [41], one of the included papers. The necessity to report datasheet or data statements was also included in papers [22,34,39].

There is no guidance in the literature specific to model development descriptions. However, papers usually describe both development and performance evaluation. The consolidated list results in the combination of main topics covering the general questions for model training, while the specific details could be found in the whole list (see Multimedia Appendix 2).

The technical validation is usually conducted by the AI model developers or providers. This procedure aims to assess the technical performance of the developed model, including model performance, reliability, robustness, and safety. The AI model is usually presented as a prototype or preproduction model before it is ready for deployment. Functional validation is typically performed as clinical trials with AI interventions and aims to assess the real-world performance, safety, usability, and relevance of the AI model in clinical use. Hence, a functional evaluation not only evaluates the AI model, but the system in which the AI model is integrated. This step could be referred to the clinical validation, a part of the AI model clinical evaluation process [53]. Before the real deployment of AI models, it is important to get evidence about the model’s safety, effectiveness, and efficiency. For this stage, the AI model should be presented as a system in an executable and ready-to-use format, so we matched this process with a deployment stage. Technical and clinical validations are crucial steps of the AI model lifecycle, and the results of this review presented the comprehensive list of reporting topics.

The section with statements could be divided into several subgroups of questions: additional information on data and model fairness; ethical considerations with protocol and registration, description of collecting the informal consent and confidential protections; contact and general details on providers, funding, and responsibilities; question to provide accessibility and reusability of the model: as code and datasets sharing, datasheet; general and technical details about model operations and monitoring. These questions correspond to the parts of critical appraisal, ethics, and reproducibility detected in [5].

### Findings Related to FAIR Principles

FAIR principles were first introduced by Wilkinson [7]. The Research Data Alliance (RDA) Data Maturity Model working group presented indicators for the FAIR assessment [16], based on the development of FAIR assessment metrics for research data [54]. The separate research group worked on the FAIR indicators for research software [55]. Furthermore, (FAIR principles for machine learning) FAIR4ML was proposed for software with machine learning [56].

Our results showed that only 9 out of 15 FAIR principles were addressed in the consolidated list. However, some papers discuss FAIR-related requirements in the description of reports. For example, the first version of the CLAIM guideline required defining the data elements [45]. Based on this reporting standard, common data elements are used by the radiological community to describe used data: a unique identifier, human-readable definition, the possible values for a particular data element, links to other ontologies (such as [Systematized Nomenclature of Medicine – Clinical Terms] SNOMED CT, [Radiology lexicon] RadLex), as well as versioning data [57,58]. These questions might meet domain-relevant community standards (R1.3) and use vocabularies that follow FAIR principles (I2). However, these questions were excluded from the recent version of the CLAIM guideline [18].

The most common questions throughout all included papers, related to FAIR principles, were about code and data availability. While it is clear for the datasets sharing, the question about code sharing was presented in different ways. Some standards require authors to deposit all code used for modeling and/or data analysis into a publicly accessible repository [18], including analytical code [17], code used to train and evaluate the model to produce all results reported in the paper [22], and access to the AI model itself [32]. Other authors mention just reproducibility [34] or state whether and how the AI intervention and/or its code can be accessed [20,21]. All in all, from this review, we see little overlap between FAIR principles and reporting standards for AI models. Our suggestion would be to apply FAIR principles (where the data is the actual model, and metadata are descriptions of the model) to AI algorithms.

### Limitations

Our systematic review has some limitations related to the evidence included. The included papers contain a few eligible scientific publications from technical databases. These papers extended the consolidated list of reporting questions with detailed information; for example, Arnold et al [41] published an extensive list of questions related to the “Statements” group. These questions might be less useful for some ML models, but we aimed to include all extracted information in the consolidated list to present the necessary aspects for reporting along the lifecycle. However, the list combined by different lifecycle stages cannot be seen as a replacement for the already existing reporting standards that are required for drafting papers and review processes.

We aligned the list with FAIR principles proposed by Wilkinson et al in 2016 [7]. In the field, there are also other known standards, such as FAIR principles for research software (FAIR4RS) [59]. However, this list does not fully include requirements for metadata, mentioning only its findability and accessibility. The specific work was done for datasets by RDA [16], aiming to adopt the general FAIR principles in the FAIR data maturity model. Another work is FAIR4ML models [56]. Both FAIR4RS as well as FAIR4ML cover principles for metadata and data (datasets or AI models) in an extended way. However, papers were published in the open-source repositories without peer review. Therefore, we decided to base our comparison on the initial FAIR principles.

During our review process, 2 authors (EA and DS) read full-text papers after the screening, including the third expert (JvS) to reach consensus, while only 1 author (EA) performed the screening in AsReview. The search was also performed by 1 author (EA); however, our search strategy was developed by 3 of the authors (EA, DS, JvS), and it was reviewed and adjusted by a librarian from the host institution. The extraction of information was performed by 1 author (EA) and presented for discussion at several group meetings and at a conference [60]. Because some of the review steps were not conducted dually and independently, we introduced some risk of error in the consolidated list of questions. Furthermore, we used the stopping criteria of 50 papers for the AsReview screening, which is enough for collecting the most relevant papers. Using this probabilistic screening may have resulted in missing a few publications. However, since our aim was not to perform a quantitative systematic review, the omission of a few publications would not influence our conclusion.

We did not evaluate the quality of reporting standards as it was done in Klement et al [47]. Some authors applied 2 following checklists: AGREE II instrument (Appraisal of Guidelines for Research and Evaluation II) [61] and RIGHT (Reporting Items for Practice Guidelines in Health care) [62], which are aiming to evaluate publications and preparing papers. We did not specify quality assessment as our main goal. Firstly, some papers were extracted from nonclinical databases where AGREE II and RIGHT were not applicable because they are designed to assess and report clinical practice guidelines. Secondly, previously published reviews [47,49] performed this quality assessment according to these 2 checklists for TRIPOD+AI, DECIDE-AI, CONSORT-AI, SPIRIT-AI, MI-CLAIM (Minimum Information About Clinical AI Modeling), STARD. Nevertheless, we used 3 evidence levels and removed low-quality papers according to the eligibility criteria.

In addition, we limited publication to the English language as it is a general publication language for standards. Furthermore, these English language papers should be open access and/or Institutional Access, which excluded technical documents (like ISO [International Organization for Standardization] standards) and regulatory papers such as the AI Act [63]. Lastly, we included only general papers regardless of specific domain (such as radiomics, biology, or pathology). Although we might miss some papers with additional questions, these likely only add details of already included questions.

### Future Implications

The extracted and consolidated list of reporting questions for each AI model lifecycle stage offers significant benefits to developers, deployers, potential users, and other stakeholders. Firstly, the consolidated list summarizes all questions from different published reporting standards. It allows for an overview of all aspects that should be reported at different stages of the AI lifecycle. Therefore, it provides transparency of lifecycle stages and limits possible challenges in later stages [51]. This aspect opens the perspective of developing the tooling to provide necessary information at the certain stage, as it was proposed by Brereton et al [64]. Future work would be to identify correspondence between our consolidated list and requirements from technical (eg, ISO standards) and regulatory (eg, the Medical Device Regulation regulation, AI Act) documents. For example, the AI Act proposes the minimal content to describe general characteristics of the AI system [63]. By using the consolidated list as AI model metadata, developers and deployers can disseminate AI models more easily than with a plain-text description [65]. Potential users could meet their needs in choosing AI models for their specific clinical tasks. However, it should be noted that the value of sharing should outweigh the effort required to create metadata. For this purpose, LLM agents could help us to create structured metadata from published papers with an AI model description. The future implications of our results should also meet the criteria for reproducibility and reusability. For that purpose, we included research questions related to FAIR principles.

The importance of adopting FAIR principles for AI has been raised before [66]. To address one of the basic aspects of the FAIR principles, metadata should be machine-readable. The Mobilized Computable Biomedical Knowledge community already proposed to use RDF and Semantic Web standards [65]. Furthermore, Adhikari et al [67] proposed to use the Web Ontology Language for explainable AI solutions by providing rich, machine-readable metadata for AI system descriptions (types of input data, model type, and task type) and parameters of use cases. We hypothesize that incorporation of FAIR principles into the consolidated list from this work will help to develop a comprehensive “digital object” description and machine actionable AI model, to speed-up the turnaround time from data analysis and AI development towards clinical practice.

### Conclusion

Our study provides the complete consolidated list of reporting questions for AI models in health care. We extracted 134 questions along the lifecycle stages as problem identification, model development, technical and functional validation, operation and monitoring, and general statements. Results showed that only 9 out of 15 general FAIR principles were addressed in the consolidated list. This consolidated list could be used to create AI model metadata to emphasize the sharing of AI models. Incorporating FAIR principles in AI models’ metadata may improve their reproducibility and reusability.

## Supplementary material

10.2196/92329 Multimedia Appendix 1 Search strategy.

10.2196/92329 Multimedia Appendix 2 The consolidated table with reporting questions.

10.2196/92329 Checklist 1 PRISMA 2020 checklist.

## Abbreviations

AGREE II: Appraisal of Guidelines for Research and Evaluation II
AsReview: Automated Systematic Review software
BERT: Bidirectional Encoder Representations from Transformers
CHEERS-AI: Consolidated Health Economic Evaluation Reporting Standards for Interventions that use AI
CLAIM: Clinical AI Modeling
CONSORT-AI: Consolidated Standards of Reporting Trials–AI
DECIDE-AI: Developmental and Exploratory Clinical Investigations of Decision Support Systems Driven by AI
DICOM: Digital Imaging and Communications in Medicine
FAIR: findable, accessible, interoperable, reusable
FAIR4ML: FAIR principles for machine learning
FAIR4RS: FAIR principles for research software
FDA: Food and Drug Administration
GAMER: Generative Artificial Intelligence Tools in Medical Research
ISO: International Organization for Standardization
LLM: large language model
MI-CLAIM: Minimum Information About Clinical Artificial Intelligence Modeling
MI-CLEAR-LLM: Minimum Reporting Items for Clear Evaluation of Accuracy Reports of Large Language Models in Health care
ML: machine learning
PRISMA: Preferred Reporting Items for Systematic Reviews and Meta-Analyses
RadLex: radiology lexicon
RDA: Research Data Alliance
REFORMS: Consensus-based Recommendations for Machine-learning-based Science
RIGHT: Appraisal of Guidelines for Research and Evaluation II
SNOMED CT: Systematized Nomenclature of Medicine – Clinical Terms
SPIRIT-AI: Standard Protocol Items: Recommendations for Interventional Trials–AI
STARD: Standards for Reporting Diagnostic Accuracy
TF-IDF: term frequency-inverse document frequency
TRIPOD: Transparent Reporting of a Multivariable Prediction Model for Individual Prognosis or Diagnosis
TRIPOD+AI: Transparent Reporting of a Multivariable Prediction Model for Individual Prognosis or Diagnosis + AI
TRIPOD-LLM: Transparent Reporting of a Multivariable Prediction Model for Individual Prognosis or Diagnosis for studies using Large Language Models
TRIPOD-SRMA: Transparent Reporting of a Multivariable Prediction Model for Individual Prognosis or Diagnosis-Systematic Reviews and Meta Analysis
