# Supplementary material for: AI Model Reporting Standards in Health Care: Systematic Review and Consolidation
Source: JMIR AI. 2026 Oct 9;5:e92329. doi: 10.2196/92329 (PMC13662782; doi:10.2196/92329)
Supplement: Supplementary file 1 [file ai-v5-e92329-s001.docx]

Supplementary materials

Table 1 – Search query

| **Database** | **N** | **Search query** |
| --- | --- | --- |
| Medline | #1 | exp checklist/ or (("guid*" or "checklist*" or "protocol*" or "statement*") adj2 ("report*" or "describe*" or "description*")).ti,ab,kf. |
|  | #2 | exp Artificial intelligence/ or ("artificial intelligence" or "machine learning" or "prediction model" or "deep learning" or AI).ti,ab,kf. |
|  | #3 | 1 and 2 |
|  | #4 | 3 not "PRISMA".ti,ab |
| Embase | #1 | exp checklist/ or (("guid*" or "checklist*" or "protocol*" or "statement*") adj2 ("report*" or "describe*" or "description*")).ti,ab,kf |
|  | #2 | exp *"artificial intelligence"/ or ("artificial intelligence" or "machine learning" or "prediction model" or "deep learning" or AI).ti,ab,kf. |
|  | #3 | 1 and 2 |
|  | #4 | 3 not "PRISMA".ti,ab |
| Scopus | #1 | ( ( INDEXTERMS ( "guidelines as topic" OR "checklist" ) OR TITLE-ABS-KEY ( "guid*" W/2 "report*" OR "checklist*" W/2 "report*" OR "protocol" W/2 "report*" OR "statement*" W/2 "report*" ) ) OR ( INDEXTERMS ( "guidelines as topic" OR "checklist" ) OR TITLE-ABS-KEY ( "guid*" W/2 "describe*" OR "checklist*" W/2 "describe*" OR "protocol" W/2 "describe*" OR "statement*" W/2 "describe*" ) ) OR ( INDEXTERMS ( "guidelines as topic" OR "checklist" ) OR TITLE-ABS-KEY ( "guid*" W/2 "description*" OR "checklist*" W/2 "description*" OR "protocol" W/2 "description*" OR "statement*" W/2 "description*" ) ) ) AND ( INDEXTERMS ( "artificial intelligence" ) OR TITLE-ABS-KEY ( "AI" OR "artificial intelligence" OR "machine learning" OR "prediction model" OR "deep learning" ) ) AND NOT TITLE-ABS-KEY ( prisma ) |
| Web of Science | #1 | (KP=("artificial intelligence")) OR TI=("artificial intelligence") OR TI=("AI") OR TI=("machine learning") OR TI=("prediction model") OR TI=("deep learning") OR AB=("artificial intelligence") OR AB=("AI") OR AB=("machine learning") OR AB=("prediction model") OR AB=("deep learning") |
|  | #2 | (KP=(guidelines) OR KP=(checklist)) OR TI=(("guid*" NEAR/2 "report*") OR ("checklist*" NEAR/2 "report*") OR ("protocol" NEAR/2 "report*") OR ("statement*" NEAR/2 "report*" )) OR TI=(("guid*" NEAR/2 "describe*") OR ("checklist*" NEAR/2 "describe*") OR ("protocol" NEAR/2 "describe*") OR ("statement*" NEAR/2 "describe*")) OR TI=(("guid*" NEAR/2 "description*") OR ("checklist*" NEAR/2 "description*") OR ("protocol" NEAR/2 "description*") OR ("statement*" NEAR/2 "description*" )) OR AB=(("guid*" NEAR/2 "report*") OR ("checklist*" NEAR/2 "report*") OR ("protocol" NEAR/2 "report*") OR ("statement*" NEAR/2 "report*" )) OR AB=(("guid*" NEAR/2 "describe*") OR ("checklist*" NEAR/2 "describe*") OR ("protocol" NEAR/2 "describe*") OR ("statement*" NEAR/2 "describe*")) OR AB=(("guid*" NEAR/2 "description*") OR ("checklist*" NEAR/2 "description*") OR ("protocol" NEAR/2 "description*") OR ("statement*" NEAR/2 "description*")) |
|  | #3 | #1 AND #2 |
|  | #4 | #3 NOT "PRISMA" |
| XEEE | #1 | ((("IEEE Terms":Standardization) OR ("Document Title":checklist) OR ("Abstract":checklist) OR ("Document Title":guideline*) OR ("Abstract":guideline*) OR ("Document Title":protocol) OR ("Abstract":protocol) OR ("Document Title":statement) OR ("Abstract":statement) AND (("Document Title":report*) OR ("Abstract":report*) OR ("Document Title":describ*) OR ("Abstract": describ*) OR ("Document Title":descript*) OR ("Abstract": descript*)) )) |
|  | #2 | ("IEEE Terms":Artificial Intelligence ) OR ("Document Title":Artificial Intelligence ) OR ("Abstract":Artificial Intelligence ) OR ("Document Title":AI) OR ("Abstract":AI) OR ("Document Title":machine learning) OR ("Abstract":machine learning) OR ("Document Title":prediction model) OR ("Abstract":prediction model) OR ("Document Title":deep learning) OR ("Abstract":deep learning) |
|  | #3 | #1 AND #2 |
| ACM digital library | #1 | [[Title: ai] OR [Title: "artificial intelligence"] OR [Title: "machine learning"] OR [Title: "prediction model"] OR [Title: "deep learning"]] AND [[Abstract: ai] OR [Abstract: "artificial intelligence"] OR [Abstract: "machine learning"] OR [Abstract: "prediction model"] OR [Abstract: "deep learning"]] AND [[Title: checklist] OR [Title: guideline*] OR [Title: protocol] OR [Title: statement*] OR [Title: report*] OR [Title: describe*] OR [Title: descript*] OR [Title: standard*]] AND [[Abstract: checklist] OR [Abstract: guideline*] OR [Abstract: protocol] OR [Abstract: statement] OR [Abstract: report*] OR [Abstract: describe*] OR [Abstract: descript*] OR [Abstract: standard*]] AND [E-Publication Date: (01/01/2016 TO 31/12/2024)] |
